# A Rare Incidence of Breakage of tip of Micropituitary Forceps during Percutaneous Discectomy - How to Remove it: A Case Report

**DOI:** 10.5704/MOJ.1511.009

**Published:** 2015-11

**Authors:** M Sureisen, BB Tan, YY Teo, CC Wong

**Affiliations:** Department of Orthopaedic Surgery, Sarawak General Hospital, Kuching, Malaysia; * Department of Orthopaedic Surgery, University Malaysia Sarawak, Kota Samarahan, Malaysia

**Keywords:** Broken micropituitary forceps tip, percutaneous discectomy, removal

## Abstract

Breakage of the tip of the micropituitary forceps during spine surgery is a rare occurrence. Retrieval of the broken tip could be a challenge in minimally invasive surgeries due to limitation of access and retrieval instruments. We describe our experience in handling such a situation during percutaneous radiofrequency discectomy. The removal was attempted, without converting into open surgery, by utilising percutaneous endoscopic lumbar discectomy working cannula and guided by image intensifier. We were able to remove the fragment without any significant morbidity to the patient. This technique for removal has not been reported previously in the literature.

## Introduction

Breakage of the tip of the micropituitary forceps during spine procedures is rare and can be implicated with medico legal litigations^1^. Retrieval of the broken fragment is more feasible in conventional spine surgeries like open discectomies. However, in minimally invasive surgeries like percutaneous disc decompression procedures, it is technically challenging because of the limitation of access and retrieval instruments.

## Case Report

Mr. KAL presented one year ago with chief complaint of lower back pain with mild radiation to left L5 dermatome. His back pain was more severe and disturbed his sleep. As he was a computer technician, he was unable to sit long in front of computer to complete his tasks.

He was involved in a motor vehicle accident 10 years prior to presentation and had sustained stable T5 compression fracture, which had been treated conservatively and healed well. He presented four years ago with a similar complaint of left L5 radiculopathy. MRI showed degenerative disc disease at L4/L5 and L5/S1 discs. He underwent back strengthening exercises, pharmacological therapy with cessation of cigarette smoking which improved his symptoms and surgery was avoided.

**Fig. 1:**
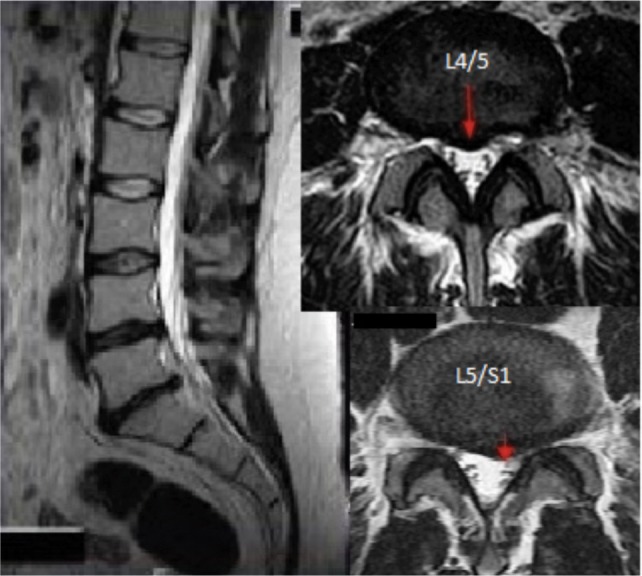
Sagittal and axial images showing central bulges and paracentral bulge at L4/L5 and L5/S1 vertebrae respectively.

On examination during the presentation a year ago, there was tenderness over the midline of his lower back but no lower limb weakness or sensory loss. Repeated MRI showed central and paracentral disc bulge at L4/L5 and L5/S1 with mild foraminal stenosis (Figure 1). After discussion, he consented to undergo two levels percutaneous radio-frequency discectomy.

Patient was placed in prone position for the procedure under local anaesthesia. Procedural steps as recommended by the manufacturer (Disk-FX^®^-Elliquence Innovative Medical Solution) were adhered to. Insertion of spinal needle, guide wire, 3.0mm cannula with dilator and trephine for annulotomy were done under fluoroscopy. Cannula was further advanced to be positioned over the posterior third of the disk. A 2.7mm micropituitary forceps was introduced via the cannula and mechanical discectomy performed.

**Fig. 2:**
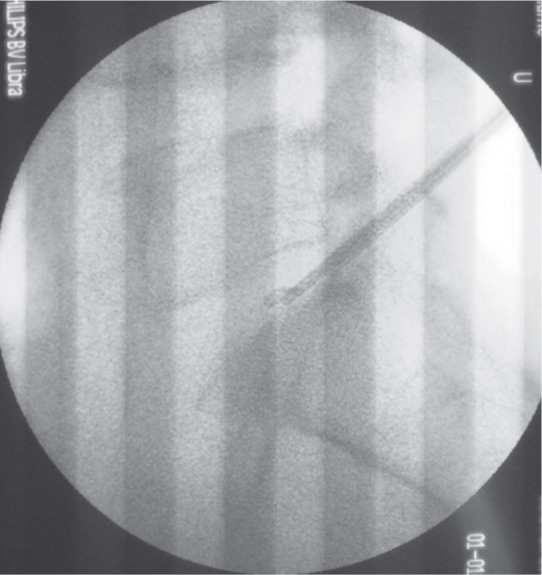
Inability to remove the broken micropituitary tip with via 3.0mm Disc-FX^®^ cannula.

However the lower jaw of the micropituitary had broken during the discectomy. Multiple attempts at removal of the tip via using a new micropituitary forceps were fruitless. We were able to grasp the blunt tip but unable to remove it out through the small diameter of the cannula (Figure 2). We realized the risk of injury to the nerve root and dural tube was higher with this blind maneuver.

**Fig. 3:**
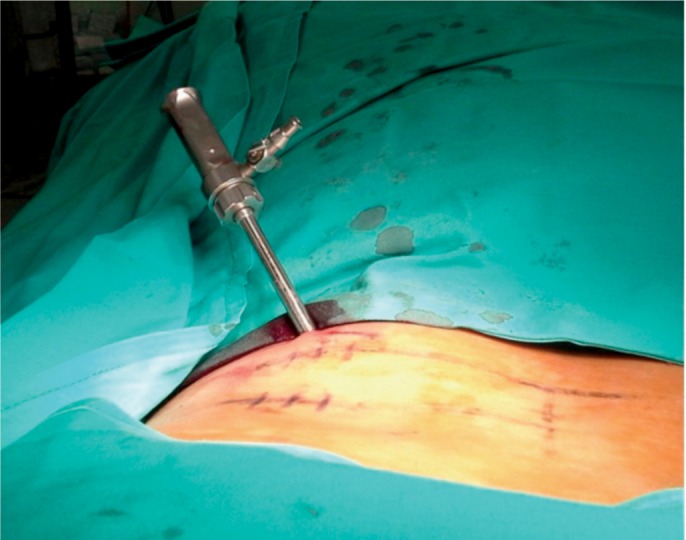
PELD working cannula with 6.8mm inner diameter – inserted under fluoroscopic guidance.

The options available were either to perform an open procedure or to attempt to retrieve using a larger working cannula. We used PELD system (Arthro Kinetics Plc) as the working cannula is wider (Inner diameter 6.8mm). After insertion of guide wire, guiding rod and sequential dilators, the working cannula was advanced near to the fragment under fluoroscopy (Figure 3).

After a few attempts, we managed to grip the fragment firmly and pull it out through the working cannula (Figure 4). The patient was awake and, cooperative throughout this procedure and was asked to indicate if painful. The broken micropituitary forceps and the tip were sent to the manufacture for assessment (Figure 4A-C). The patient had mild left L5 dermatomal radicular pain post-procedure but resolved completely after three months. Improvement of JOA Back Pain Evaluation Questionnaire (JOABPEQ)^2^ from 78 to 94 was noted. He was prescribed Gabapentin for relief of the radiculopathy. His back pain also improved and he was able to return to his previous work.

**Fig. 4a:**
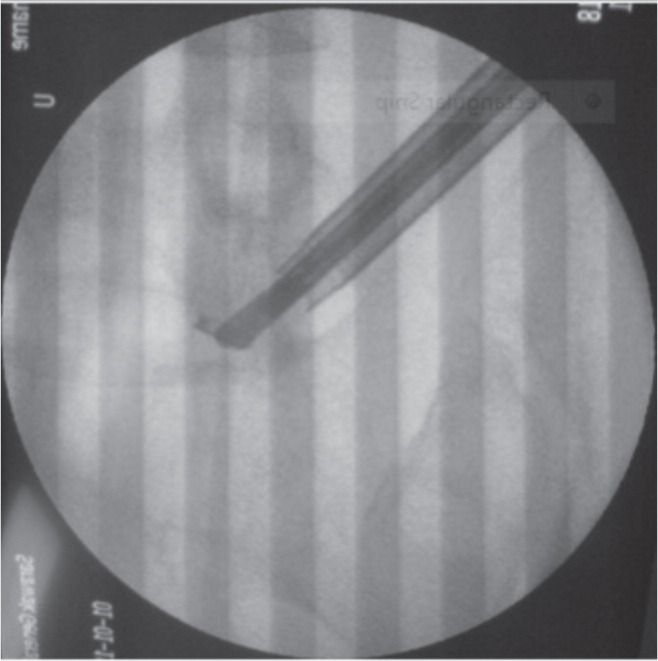
Broken fragment was gripped firmly with a new micropituitary.

**Fig. 4b:**
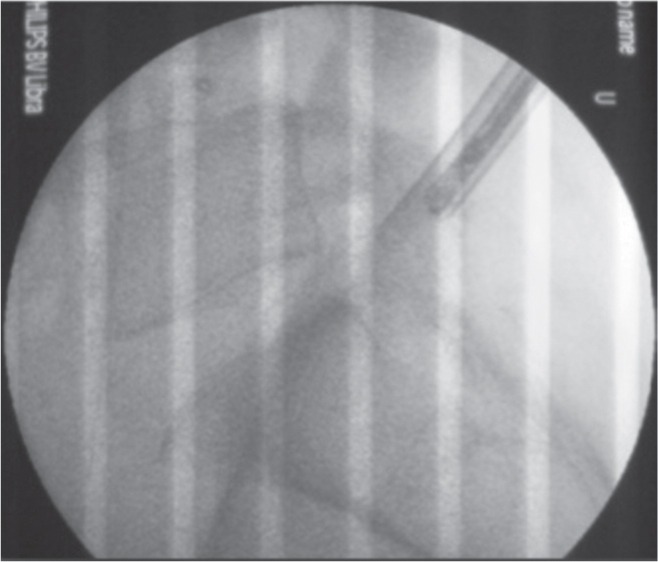
Broken jaw was pulled out through the working cannula.

**Fig. 4c:**
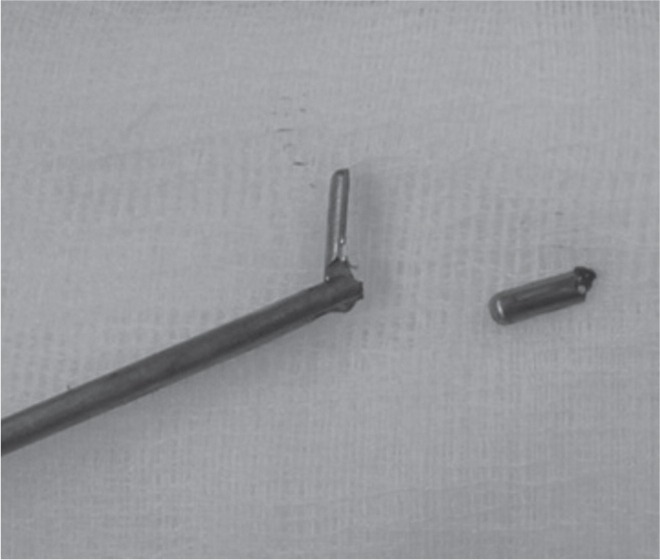
The micropituitary with broken lower jaw.

## Discussion

Breakage of the tip of the micropituitary forceps is rare during spine surgery. The reasons could vary from normal wear and tear, applying excessive pressure while handling the instrument, product defect or inappropriate maintenance by operating theatre personnel. Although rare, it has significant medicolegal implications. At least four medical instrument manufacturers have reported breakage of the tip of their instrument^3,4,5^.

This problem is obviously easier to handle in open spine surgeries. However, in minimally invasive surgeries, especially percutaneous surgeries, we are limited by the adjacent anatomical structures like iliac crest, narrowed intervertebral foramen and intervertebral disc, as well as facet joint hypertrophy. If the fragment is not retrievable, more aggressive discectomy might be required, with patient counselled regarding the additional procedure under general anaesthesia. This could lead to conflict in doctor-patient relationship and litigations.

We hope the technique described here will negate these consequences. By using a larger working cannula (PELD cannula), the broken fragment can be easily removed under flouroscopy. The broken fragment should be manipulated into the disc space to prevent injury to nerve tissue in the epidural space.

We suggest the alternate usage of endoscope/foraminoscope if there is difficulty in retrieving this fragment. With direct visualization via an endoscope, supplemented with flouroscopy, the task will be successful.

## Conclusion

PELD equipment is very handy in the retrieval of small metal part of a broken instrument. It can be done safely under local anesthetic with mild intravenous sedation.
